# Crystal structure and electrical resistance property of Rb_0.21_(H_2_O)_*y*_WS_2_

**DOI:** 10.1107/S2056989019007941

**Published:** 2019-06-11

**Authors:** Yuanlv Mao, Yuqiang Fang, Dong Wang, Kejun Bu, Sishun Wang, Wei Zhao, Fuqiang Huang

**Affiliations:** a State Key Laboratory of High Performance Ceramics and Superfine Microstructure, Shanghai Institute of Ceramics, Chinese Academy of Sciences, Shanghai 200050, People’s Republic of China; b School of Physical Science and Technology, ShanghaiTech University, Shanghai 201210, People’s Republic of China

**Keywords:** crystal structure, quasi two-dimensional structure, disorder, electric properties

## Abstract

Rb_0.21_(H_2_O)_*y*_WS_2_, rubidium hydrate di­thio­tungstate, is a new quasi two-dimensional sulfide. Its crystal structure consists of ordered WS_2_ layers, separated by disordered Rb^+^ ions and water mol­ecules. All atomic sites are located on mirror planes. The WS_2_ layers are composed of edge-sharing [WS_6_] octa­hedra and extend parallel to (001). The presence of structural water was revealed by thermogravimetry, but the position and exact amount could not be determined in the present study. The temperature dependence of the electrical resistance indicates that Rb_0.21_(H_2_O)_*y*_WS_2_ is semiconducting between 80–300 K.

## Abstract 2

Determination of the crystal structure of Rb_0.21_(H_2_O)_*y*_WS_2_ is beneficial to understanding the topotactic reaction process to form metastable WS_2_. The temperature dependence of the electrical resistance indicates that Rb_0.21_(H_2_O)_*y*_WS_2_ is semiconducting at 80-300 K.

## Chemical context   

Typical two-dimensional structures of *M*S_2_ compounds (*M* = transition metals of group IVB–VIB) facilitate the inter­calation of various atoms, ions or organic mol­ecules (Whittingham *et al.*, 1978 ▸). For example, *A*_x_ *M*S_2_ (*A* = alkali metal; *M* = Nb, Ta, Ti, V) compounds can be prepared in high-temperature solid-state reactions (800–1000 K). These com­pounds can react with water mol­ecules to form ionic hydrates *A* ^+^_*x*_(H_2_O)_*y*_[*M*S_2_]^*x*−^ (Omloo & Jellinek, 1970 ▸; Lerf & Schöllhorn, 1977 ▸; Lobert *et al.*, 1992 ▸) that exhibit ion-exchange and solvent-exchange capacities. Some of the *A* ^+^_*x*_(H_2_O)_*y*_[*M*S_2_]^*x*−^ compounds show unusual superconducting properties (Schöllhorn & Weiss, 1974 ▸; Sernetz *et al.*, 1974 ▸). Recently, by removing alkali ions from inter­calated *A* ^+^_*x*_(H_2_O)_*y*_[*M*S_2_]^*x*−^ (*A* = alkali metal) compounds, several metastable *M*S_2_ (*M* = Mo, W) phases with new crystal structures and novel physical properties were reported (Fang *et al.*, 2018 ▸, 2019 ▸). In order to identify the formation mechanism of metastable *M*S_2_ from *A* ^+^_*x*_(H_2_O)_*y*_[*M*S_2_]^*x*−^, it is necessary to uncover the role of alkali ions inter­calated into the inter­layers of *M*S_2_.

In this communication, we report the preparation of Rb_0.21_(H_2_O)_*y*_WS_2_, its crystal structure determination by single crystal X-ray diffraction, its thermal behaviour and its electrical resistance property.

## Structural commentary   

**Figure 1:**
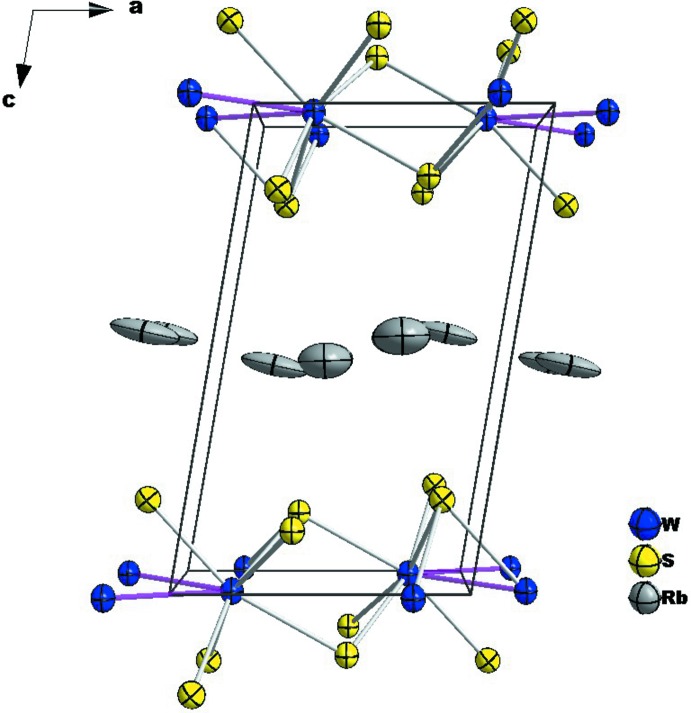
Crystal structure of Rb_0.21_(H_2_O)_*y*_WS_2_ with displacement ellipsoids drawn at the 30% probability level.

Rb_0.21_(H_2_O)_*y*_WS_2_ crystallizes in the monoclinic *P*2_1_/m (No. 11) space group. The structure consists of one independent W site, two independent S sites and two independent Rb sites, all of them located on a mirror plane (Wyckoff position 2*e*). The crystal structure features ordered WS_2_ layers separated by disordered Rb^+^ ions, and of water mol­ecules. The latter could not be localized in the current study, hence *y* in Rb_0.21_(H_2_O)_*y*_WS_2_ remains undetermined (see *Experimental*, and discussion below). Compared with [WS_6_]^8–^ trigonal prisms in 2H-WS_2_ (Schutte *et al.*, 1987 ▸), the WS_2_ layer in Rb_0.21_(H_2_O)_*y*_WS_2_ is composed of edge-sharing [WS_6_]^8.21–^ octa­hedra. The W—S bond lengths range from 2.403 (4) Å to 2.550 (5) Å, and thus the average W—S distance is larger than that in 2H-WS_2_ [2.405 (5) Å; Schutte *et al.*, 1987 ▸]. The WS_2_ layers extend parallel to (001) (Fig. 1 ▸). The shortest W—W bond length of 2.7678 (15) Å is between pairs of W atoms aligned in the [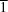
10] direction, much shorter than the W⋯W distance of 3.2524 (18) Å along [010]. Similar metal–metal separations also exist in some metastable *M*S_2_ phases prepared by de-inter­calating alkali ions from *A*_x_(H_2_O)_y_ *M*S_2_ compounds (Yu *et al.*, 2018 ▸; Shang *et al.*, 2018 ▸). The Rb^+^ cations show a one-sided coordination to the S atoms of the adjacent layer. The Rb—S bonds range from 3.47 (7) Å to 3.64 (5) Å, comparable to the Rb—S bonds [3.344 (7)–3.561 (1) Å] in RbCr_5_S_8_ (Huster, 1978 ▸).

Similar to K_*x*_(H_2_O)_*y*_TaS_2_ and K_*x*_(H_2_O)_*y*_NbS_2_ (Graf *et al.*, 1977 ▸), it was impossible to determine the light O atoms of water mol­ecules in the title compound from X-ray diffraction data at room temperature, as a result of diffuse electron density in the inter­layer space. However, we could localize the positions of disordered Rb^+^ ions with large displacement parameters. Stacking disorder of the layers is common for layered dichalcogenides, which may contribute to the diffuse electron density. Large displacement parameters of exchangeable cations and water mol­ecules were also reported for *A*_x_(H_2_O)_y_TaS_2_ and *A*_x_(H_2_O)_y_NbS_2_ (*A* = alkali metal) compounds (Röder *et al.*, 1979 ▸; Wein *et al.* 1986 ▸; Lobert *et al.*, 1992 ▸).

## Electrical resistance property   

**Figure 2:**
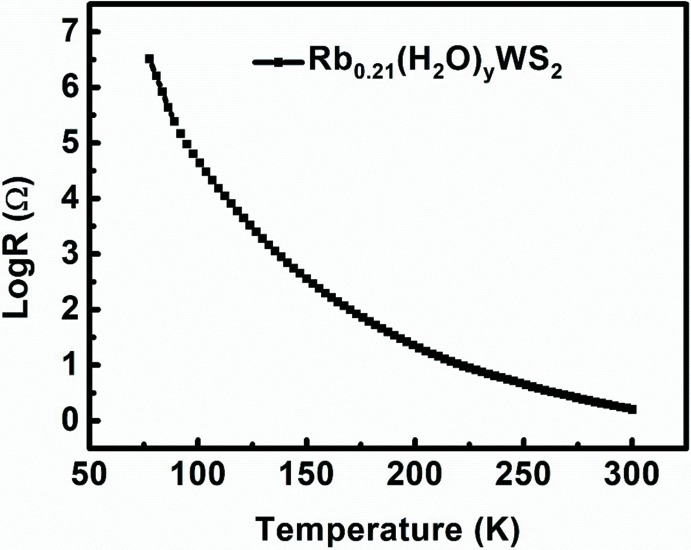
Temperature-dependence of the log(Resistance) for Rb_0.21_(H_2_O)_*y*_WS_2_.

The electrical resistance of Rb_0.21_(H_2_O)_*y*_WS_2_ increases with the decrease of temperature (80–300 K) (Fig. 2 ▸), which is characteristic of a semiconductor.

## Synthesis and crystallization   

**Figure 3:**
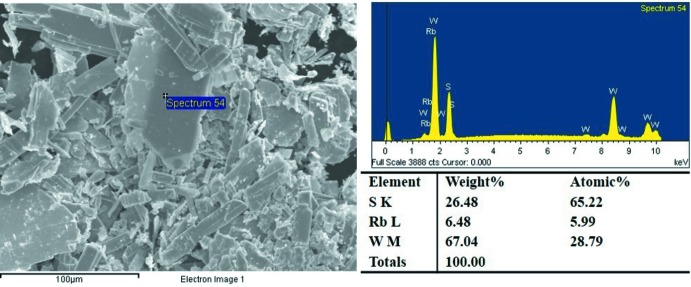
SEM image and EDXS spectrum of Rb_0.21_(H_2_O)_*y*_WS_2_.

**Figure 4:**
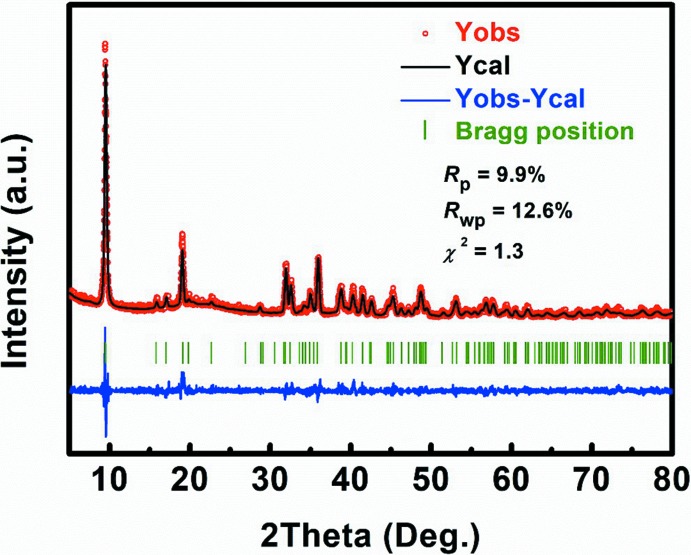
Rietveld plot of Rb_0.21_(H_2_O)_*y*_WS_2_.

**Figure 5:**
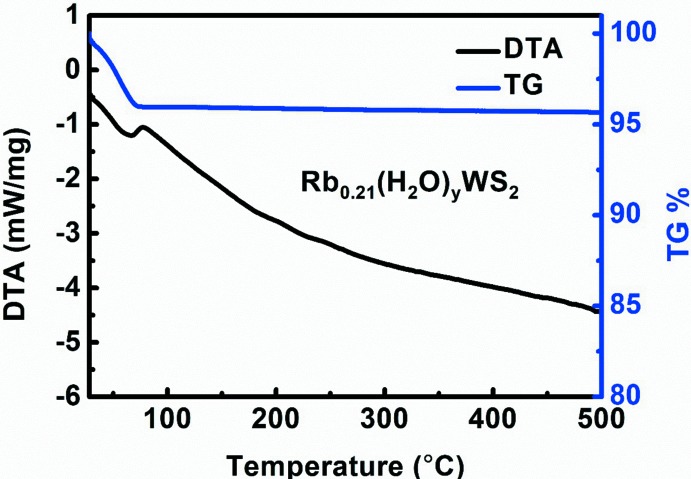
TG–DTA analysis of Rb_0.21_(H_2_O)_*y*_WS_2_.

**Figure 6:**
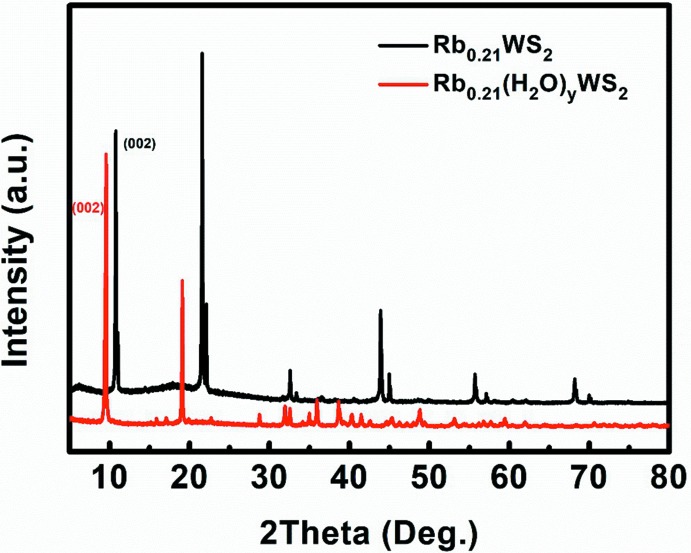
Power X-ray diffraction pattern of Rb_0.21_(H_2_O)_*y*_WS_2_ and Rb_0.21_WS_2_.

A rubidium di­thio­tungstate Rb_*x*_WS_2_ was synthesized in a solid-state reaction. The starting Rb_2_S_2_ powder was prepared in a reaction of stoichiometric amounts of Rb pieces and S powder in liquid NH_3_. The obtained Rb_2_S_2_ powder, W powder and S powder were mixed in the molar ratio of 1:1:1 in a glove box filled with Ar. The mixture was ground carefully and loaded in a carbon-coated fused-silica tube. The tube was sealed under a 10^−4^ Torr atmosphere and slowly heated to 1123 K at 5 K min^−1^. After three days, the furnace was cooled down naturally to room temperature. Subsequent removal of the extra flux by washing with distilled water led to the isolation of crystals of Rb_0.21_(H_2_O)_*y*_WS_2_. The morphology and element composition were investigated by using an EDXS-equipped Hitachi S-4800 scanning electronic microscope. In addition, the Rb/W ratio in the Rb_x_(H_2_O)_y_WS_2_ crystals was determined by ICP-OES. The SEM image and EDX spectrum of Rb_0.21_(H_2_O)_y_WS_2_ crystals are shown in Fig. 3 ▸. The ratio of Rb/W from the EDXS analysis is close to 0.21, which is consistent with the the diffraction data and results from ICP–OES measurements (Table 1 ▸). The experimental powder X-ray diffraction (PXRD) pattern matches well with the simulated one (Fig. 4 ▸) by using the Rietveld refinement method (Rodríguez-Carvajal, 1993 ▸; *R*_p_ = 9.9%, *R*_wp_ = 12.6% and *χ* ^2^ = 1.3). In the TG–DTA analyses (Fig. 5 ▸), one obvious endothermic effect and concomitant mass loss were observed at 343 K, which is associated with water evaporation. In order to judge whether water mol­ecules are surface-adsorbed water or structural water, the Rb_0.21_(H_2_O)_*y*_WS_2_ crystals were heated up to 373 K for further PXRD measurement. The sample was prepared in an Ar-protected glove box and sealed with vacuum tape. The (002) reflection clearly moved to higher diffraction angles, indicating the shrinkage of the unit cell due to loss of inter­calated water (Fig. 6 ▸). However, it was impossible to accurately determine the water content by mass loss alone because of the inter­ference of possible surface-adsorbed water.

## Refinement details   

Crystal data, data collection and structure refinement details are summarized in Table 2 ▸. The localization of ordered W and S sites of the WS_2_ layers was unproblematic. The highest inter­layer difference electron density peak was then treated as a single but partially occupied Rb site. No evidence of superstructure reflections in reciprocal space was found for the ordering of the Rb site. Then, the W, S sites and the underoccupied Rb site were refined with anisotropic displacement parameters. Because of very large anisotropic displacement parameters (*U* ^11^ = 0.59 Å^2^) of the Rb site, splitting of this site was considered, resulting in a residual *R*_1_ = 0.051. Modelling the O sites as being part of this disorder, or of remaining electron density peaks in the vicinity of the Rb sites was not successful, and therefore we did not include the apparently disordered water mol­ecules in the final structure model. The remaining maximum and minimum electron densities are located 0.87 and 1.14 Å, respectively, from the W1 site.

## Supplementary Material

Crystal structure: contains datablock(s) I. DOI: 10.1107/S2056989019007941/wm5498sup1.cif

Structure factors: contains datablock(s) I. DOI: 10.1107/S2056989019007941/wm5498Isup2.hkl

CCDC reference: 1920386

Additional supporting information:  crystallographic information; 3D view; checkCIF report

## Figures and Tables

**Table 1:** Results of ICP–OES measurement of Rb_0.21_(H_2_O)_*y*_WS_2_

Element	Weight (%)	atom (%)
W	67.6	36.77
Rb	6.6	7.72

**Table 2:** Experimental details

Crystal data	
Chemical formula	Rb_0.21_(H_2_O)_*y*_WS_2_
*M*_r_	277.23
Crystal system, space group	Monoclinic, *P*2_1_/*m*
Temperature (K)	298
*a*, *b*, *c* (Å)	5.703 (3), 3.2524 (18), 9.423 (5)
β (°)	99.724 (16)
*V* (Å^3^)	172.27 (16)
*Z*	2
Radiation type	Mo *K*α
μ (mm^−1^)	39.25
Crystal size (mm)	0.05 × 0.03 × 0.01

Data collection	
Diffractometer	Bruker APEXII CCD
Absorption correction	Multi-scan (*SADABS*; Bruker, 2015 ▸)
*T*_min_, *T*_max_	0.251, 0.674
No. of measured, independent and observed [*I* > 2σ(*I*)] reflections	1167, 352, 327
*R*_int_	0.030
(sin θ/λ)_max_ (Å^−1^)	0.593

Refinement	
*R*[*F* ^2^ > 2σ(*F* ^2^)], *wR*(*F* ^2^), *S*	0.050, 0.124, 1.10
No. of reflections	352
No. of parameters	33
H-atom treatment	H-atom parameters not defined
Δρ_max_, Δρ_min_ (e Å^−3^)	2.45, −1.66

Computer programs: *APEX3* and *SAINT* (Bruker, 2015 ▸), *SHELXS* (Sheldrick, 2008 ▸), *SHELXL2014/7* (Sheldrick, 2015 ▸), *DIAMOND* (Brandenburg, 2004 ▸) and *publCIF* (Westrip, 2010 ▸).

## supplementary crystallographic information

### Crystal data

**Table:**

Rb_0.21_(H_2_O)yWS_2_	*F*(000) = 237
*M**_r_* = 277.23	*D*_x_ = 5.345 Mg m^−^^3^
Monoclinic, *P*2_1_/*m*	Mo *K*α radiation, λ = 0.71073 Å
*a* = 5.703 (3) Å	Cell parameters from 68 reflections
*b* = 3.2524 (18) Å	θ = 3.9–23.0°
*c* = 9.423 (5) Å	µ = 39.25 mm^−^^1^
β = 99.724 (16)°	*T* = 298 K
*V* = 172.27 (16) Å^3^	Plate, black
*Z* = 2	0.05 × 0.03 × 0.01 mm

### Data collection

**Table:**

Bruker APEXII CCD diffractometer	327 reflections with *I* > 2σ(*I*)
phi and ω scans	*R*_int_ = 0.030
Absorption correction: multi-scan (*SADABS*; Bruker, 2015)	θ_max_ = 24.9°, θ_min_ = 2.2°
*T*_min_ = 0.251, *T*_max_ = 0.674	*h* = −6→6
1167 measured reflections	*k* = −3→3
352 independent reflections	*l* = −11→10

### Refinement

**Table:**

Refinement on *F*^2^	0 restraints
Least-squares matrix: full	H-atom parameters not defined
*R*[*F*^2^ > 2σ(*F*^2^)] = 0.050	*w* = 1/[σ^2^(*F*_o_^2^) + (0.1024*P*)^2^] where *P* = (*F*_o_^2^ + 2*F*_c_^2^)/3
*wR*(*F*^2^) = 0.124	(Δ/σ)_max_ < 0.001
*S* = 1.10	Δρ_max_ = 2.45 e Å^−^^3^
352 reflections	Δρ_min_ = −1.66 e Å^−^^3^
33 parameters	

### Special details

**Table:**

Geometry. All esds (except the esd in the dihedral angle between two l.s. planes) are estimated using the full covariance matrix. The cell esds are taken into account individually in the estimation of esds in distances, angles and torsion angles; correlations between esds in cell parameters are only used when they are defined by crystal symmetry. An approximate (isotropic) treatment of cell esds is used for estimating esds involving l.s. planes.

### Fractional atomic coordinates and isotropic or equivalent isotropic displacement parameters (Å^2^)

**Table:**

	*x*	*y*	*z*	*U*_iso_*/*U*_eq_	Occ. (<1)
W1	0.19782 (11)	0.7500	1.00615 (8)	0.0371 (5)	
S2	0.3744 (9)	0.2500	0.8600 (6)	0.0376 (12)	
S3	0.1409 (10)	0.2500	1.1850 (6)	0.0401 (12)	
Rb4	0.21 (4)	−0.2500	0.534 (6)	0.14 (6)	0.14 (7)
Rb5	0.38 (2)	−0.2500	0.525 (8)	0.17 (2)	0.20 (6)

### Atomic displacement parameters (Å^2^)

**Table:**

	*U*^11^	*U*^22^	*U*^33^	*U*^12^	*U*^13^	*U*^23^
W1	0.0319 (7)	0.0374 (7)	0.0423 (7)	0.000	0.0073 (4)	0.000
S2	0.034 (2)	0.038 (3)	0.041 (3)	0.000	0.007 (2)	0.000
S3	0.041 (3)	0.039 (3)	0.041 (3)	0.000	0.008 (2)	0.000
Rb4	0.28 (14)	0.12 (4)	0.04 (2)	0.000	0.06 (4)	0.000
Rb5	0.18 (6)	0.24 (6)	0.08 (3)	0.000	0.01 (3)	0.000

### Geometric parameters (Å, º)

**Table:**

W1—S3^i^	2.403 (4)	Rb4—Rb5	0.99 (10)
W1—S3	2.403 (4)	Rb4—Rb4^x^	2.9 (3)
W1—S3^ii^	2.408 (5)	Rb4—Rb4^xi^	2.9 (3)
W1—S2	2.454 (4)	Rb4—Rb5^xii^	3.0 (3)
W1—S2^i^	2.454 (4)	Rb4—Rb5^xiii^	3.0 (3)
W1—S2^iii^	2.550 (5)	Rb4—Rb4^i^	3.252 (2)
W1—W1^ii^	2.7678 (15)	Rb4—Rb4^v^	3.2524 (19)
W1—W1^iv^	2.7678 (15)	Rb4—Rb5^i^	3.40 (3)
S2—W1^v^	2.454 (4)	Rb4—Rb5^v^	3.40 (3)
S2—W1^iii^	2.550 (5)	Rb4—S2^v^	3.47 (7)
S2—Rb4	3.47 (7)	Rb4—S3^vii^	3.58 (11)
S2—Rb4^i^	3.47 (7)	Rb5—Rb5^xii^	2.23 (19)
S2—Rb5^i^	3.56 (8)	Rb5—Rb5^xiii^	2.23 (19)
S2—Rb5	3.56 (8)	Rb5—Rb4^xii^	3.0 (3)
S3—W1^v^	2.403 (4)	Rb5—Rb4^xiii^	3.0 (3)
S3—W1^ii^	2.408 (5)	Rb5—Rb5^i^	3.2524 (18)
S3—Rb5^vi^	3.53 (6)	Rb5—Rb5^v^	3.2524 (18)
S3—Rb4^vii^	3.58 (11)	Rb5—Rb4^v^	3.40 (3)
S3—Rb4^viii^	3.63 (4)	Rb5—Rb4^i^	3.40 (3)
S3—Rb4^ix^	3.63 (4)	Rb5—S3^vi^	3.53 (6)
S3—Rb5^viii^	3.64 (5)	Rb5—S2^v^	3.56 (8)
S3—Rb5^ix^	3.64 (5)		
			
S3^i^—W1—S3	85.18 (18)	Rb5^xii^—Rb4—Rb5^i^	40 (3)
S3^i^—W1—S3^ii^	109.76 (14)	Rb5^xiii^—Rb4—Rb5^i^	106 (4)
S3—W1—S3^ii^	109.76 (14)	Rb4^i^—Rb4—Rb5^i^	16.9 (16)
S3^i^—W1—S2	163.5 (2)	Rb4^v^—Rb4—Rb5^i^	163.1 (16)
S3—W1—S2	93.55 (13)	Rb5—Rb4—Rb5^v^	73.1 (16)
S3^ii^—W1—S2	86.20 (17)	Rb4^x^—Rb4—Rb5^v^	71 (3)
S3^i^—W1—S2^i^	93.55 (13)	Rb4^xi^—Rb4—Rb5^v^	140 (7)
S3—W1—S2^i^	163.5 (2)	Rb5^xii^—Rb4—Rb5^v^	106 (4)
S3^ii^—W1—S2^i^	86.20 (17)	Rb5^xiii^—Rb4—Rb5^v^	40 (3)
S2—W1—S2^i^	82.99 (18)	Rb4^i^—Rb4—Rb5^v^	163.1 (16)
S3^i^—W1—S2^iii^	83.36 (18)	Rb4^v^—Rb4—Rb5^v^	16.9 (16)
S3—W1—S2^iii^	83.36 (18)	Rb5^i^—Rb4—Rb5^v^	146 (3)
S3^ii^—W1—S2^iii^	161.7 (2)	Rb5—Rb4—S2^v^	87 (10)
S2—W1—S2^iii^	80.13 (16)	Rb4^x^—Rb4—S2^v^	91.3 (12)
S2^i^—W1—S2^iii^	80.13 (16)	Rb4^xi^—Rb4—S2^v^	124 (4)
S3^i^—W1—W1^ii^	102.78 (13)	Rb5^xii^—Rb4—S2^v^	108 (5)
S3—W1—W1^ii^	54.97 (13)	Rb5^xiii^—Rb4—S2^v^	79 (2)
S3^ii^—W1—W1^ii^	54.79 (10)	Rb4^i^—Rb4—S2^v^	118.0 (6)
S2—W1—W1^ii^	89.78 (11)	Rb4^v^—Rb4—S2^v^	62.0 (6)
S2^i^—W1—W1^ii^	140.78 (13)	Rb5^i^—Rb4—S2^v^	116 (4)
S2^iii^—W1—W1^ii^	136.53 (8)	Rb5^v^—Rb4—S2^v^	62 (2)
S3^i^—W1—W1^iv^	54.97 (13)	Rb5—Rb4—S2	87 (10)
S3—W1—W1^iv^	102.78 (13)	Rb4^x^—Rb4—S2	124 (4)
S3^ii^—W1—W1^iv^	54.79 (10)	Rb4^xi^—Rb4—S2	91.3 (12)
S2—W1—W1^iv^	140.78 (13)	Rb5^xii^—Rb4—S2	79 (2)
S2^i^—W1—W1^iv^	89.78 (11)	Rb5^xiii^—Rb4—S2	108 (5)
S2^iii^—W1—W1^iv^	136.53 (8)	Rb4^i^—Rb4—S2	62.0 (6)
W1^ii^—W1—W1^iv^	71.96 (5)	Rb4^v^—Rb4—S2	118.0 (6)
W1^v^—S2—W1	82.99 (18)	Rb5^i^—Rb4—S2	62 (2)
W1^v^—S2—W1^iii^	99.87 (16)	Rb5^v^—Rb4—S2	116 (4)
W1—S2—W1^iii^	99.87 (16)	S2^v^—Rb4—S2	56.0 (12)
W1^v^—S2—Rb4	96.3 (15)	Rb5—Rb4—S3^vii^	138 (10)
W1—S2—Rb4	137 (3)	Rb4^x^—Rb4—S3^vii^	68 (4)
W1^iii^—S2—Rb4	122 (3)	Rb4^xi^—Rb4—S3^vii^	68 (4)
W1^v^—S2—Rb4^i^	137 (3)	Rb5^xii^—Rb4—S3^vii^	133.8 (12)
W1—S2—Rb4^i^	96.3 (15)	Rb5^xiii^—Rb4—S3^vii^	133.8 (12)
W1^iii^—S2—Rb4^i^	122 (3)	Rb4^i^—Rb4—S3^vii^	90.000 (2)
Rb4—S2—Rb4^i^	56.0 (12)	Rb4^v^—Rb4—S3^vii^	90.000 (1)
W1^v^—S2—Rb5^i^	150.1 (12)	Rb5^i^—Rb4—S3^vii^	102.5 (13)
W1—S2—Rb5^i^	105.1 (13)	Rb5^v^—Rb4—S3^vii^	102.5 (12)
W1^iii^—S2—Rb5^i^	106.8 (18)	S2^v^—Rb4—S3^vii^	56.3 (7)
Rb4—S2—Rb5^i^	57.9 (7)	S2—Rb4—S3^vii^	56.3 (7)
Rb4^i^—S2—Rb5^i^	16.1 (18)	Rb4—Rb5—Rb5^xii^	132 (4)
W1^v^—S2—Rb5	105.1 (13)	Rb4—Rb5—Rb5^xiii^	132 (4)
W1—S2—Rb5	150.1 (12)	Rb5^xii^—Rb5—Rb5^xiii^	94 (10)
W1^iii^—S2—Rb5	106.8 (18)	Rb4—Rb5—Rb4^xii^	146 (2)
Rb4—S2—Rb5	16.1 (18)	Rb5^xii^—Rb5—Rb4^xii^	14.3 (18)
Rb4^i^—S2—Rb5	57.9 (7)	Rb5^xiii^—Rb5—Rb4^xii^	80 (8)
Rb5^i^—S2—Rb5	54.4 (14)	Rb4—Rb5—Rb4^xiii^	146 (2)
W1^v^—S3—W1	85.18 (18)	Rb5^xii^—Rb5—Rb4^xiii^	80 (8)
W1^v^—S3—W1^ii^	70.24 (14)	Rb5^xiii^—Rb5—Rb4^xiii^	14.3 (18)
W1—S3—W1^ii^	70.24 (14)	Rb4^xii^—Rb5—Rb4^xiii^	66 (7)
W1^v^—S3—Rb5^vi^	111.4 (15)	Rb4—Rb5—Rb5^i^	90.00 (5)
W1—S3—Rb5^vi^	111.4 (16)	Rb5^xii^—Rb5—Rb5^i^	43 (5)
W1^ii^—S3—Rb5^vi^	178 (2)	Rb5^xiii^—Rb5—Rb5^i^	137 (5)
W1^v^—S3—Rb4^vii^	132.7 (12)	Rb4^xii^—Rb5—Rb5^i^	57 (3)
W1—S3—Rb4^vii^	132.7 (11)	Rb4^xiii^—Rb5—Rb5^i^	123 (3)
W1^ii^—S3—Rb4^vii^	94 (3)	Rb4—Rb5—Rb5^v^	90.00 (10)
Rb5^vi^—S3—Rb4^vii^	83.2 (13)	Rb5^xii^—Rb5—Rb5^v^	137 (5)
W1^v^—S3—Rb4^viii^	159 (2)	Rb5^xiii^—Rb5—Rb5^v^	43 (5)
W1—S3—Rb4^viii^	109.0 (6)	Rb4^xii^—Rb5—Rb5^v^	123 (3)
W1^ii^—S3—Rb4^viii^	129 (3)	Rb4^xiii^—Rb5—Rb5^v^	57 (3)
Rb5^vi^—S3—Rb4^viii^	49 (5)	Rb5^i^—Rb5—Rb5^v^	180.00 (7)
Rb4^vii^—S3—Rb4^viii^	47 (5)	Rb4—Rb5—Rb4^v^	73.1 (17)
W1^v^—S3—Rb4^ix^	109.0 (6)	Rb5^xii^—Rb5—Rb4^v^	153 (5)
W1—S3—Rb4^ix^	159 (2)	Rb5^xiii^—Rb5—Rb4^v^	60 (6)
W1^ii^—S3—Rb4^ix^	129 (3)	Rb4^xii^—Rb5—Rb4^v^	140 (3)
Rb5^vi^—S3—Rb4^ix^	49 (5)	Rb4^xiii^—Rb5—Rb4^v^	74 (4)
Rb4^vii^—S3—Rb4^ix^	47 (5)	Rb5^i^—Rb5—Rb4^v^	163.1 (16)
Rb4^viii^—S3—Rb4^ix^	53.3 (7)	Rb5^v^—Rb5—Rb4^v^	16.9 (16)
W1^v^—S3—Rb5^viii^	147.5 (16)	Rb4—Rb5—Rb4^i^	73.1 (16)
W1—S3—Rb5^viii^	103.9 (12)	Rb5^xii^—Rb5—Rb4^i^	60 (6)
W1^ii^—S3—Rb5^viii^	142.2 (16)	Rb5^xiii^—Rb5—Rb4^i^	153 (5)
Rb5^vi^—S3—Rb5^viii^	36 (3)	Rb4^xii^—Rb5—Rb4^i^	74 (4)
Rb4^vii^—S3—Rb5^viii^	61 (4)	Rb4^xiii^—Rb5—Rb4^i^	140 (3)
Rb4^viii^—S3—Rb5^viii^	15.7 (16)	Rb5^i^—Rb5—Rb4^i^	16.9 (16)
Rb4^ix^—S3—Rb5^viii^	55.8 (6)	Rb5^v^—Rb5—Rb4^i^	163.1 (16)
W1^v^—S3—Rb5^ix^	103.9 (12)	Rb4^v^—Rb5—Rb4^i^	146 (3)
W1—S3—Rb5^ix^	147.5 (16)	Rb4—Rb5—S3^vi^	125 (9)
W1^ii^—S3—Rb5^ix^	142.2 (16)	Rb5^xii^—Rb5—S3^vi^	75 (3)
Rb5^vi^—S3—Rb5^ix^	36 (3)	Rb5^xiii^—Rb5—S3^vi^	75 (3)
Rb4^vii^—S3—Rb5^ix^	61 (4)	Rb4^xii^—Rb5—S3^vi^	67 (4)
Rb4^viii^—S3—Rb5^ix^	55.8 (6)	Rb4^xiii^—Rb5—S3^vi^	67 (4)
Rb4^ix^—S3—Rb5^ix^	15.7 (16)	Rb5^i^—Rb5—S3^vi^	90.000 (5)
Rb5^viii^—S3—Rb5^ix^	53.0 (8)	Rb5^v^—Rb5—S3^vi^	90.000 (2)
Rb5—Rb4—Rb4^x^	141 (2)	Rb4^v^—Rb5—S3^vi^	100 (3)
Rb5—Rb4—Rb4^xi^	141 (2)	Rb4^i^—Rb5—S3^vi^	100 (3)
Rb4^x^—Rb4—Rb4^xi^	69 (9)	Rb4—Rb5—S2^v^	77 (8)
Rb5—Rb4—Rb5^xii^	34 (2)	Rb5^xii^—Rb5—S2^v^	128 (3)
Rb4^x^—Rb4—Rb5^xii^	157 (2)	Rb5^xiii^—Rb5—S2^v^	87 (3)
Rb4^xi^—Rb4—Rb5^xii^	107.5 (14)	Rb4^xii^—Rb5—S2^v^	123 (3)
Rb5—Rb4—Rb5^xiii^	34 (2)	Rb4^xiii^—Rb5—S2^v^	92.3 (10)
Rb4^x^—Rb4—Rb5^xiii^	107.5 (13)	Rb5^i^—Rb5—S2^v^	117.2 (7)
Rb4^xi^—Rb4—Rb5^xiii^	157 (2)	Rb5^v^—Rb5—S2^v^	62.8 (7)
Rb5^xii^—Rb4—Rb5^xiii^	66 (7)	Rb4^v^—Rb5—S2^v^	59.7 (19)
Rb5—Rb4—Rb4^i^	90.00 (5)	Rb4^i^—Rb5—S2^v^	112 (3)
Rb4^x^—Rb4—Rb4^i^	125 (5)	S3^vi^—Rb5—S2^v^	55.4 (8)
Rb4^xi^—Rb4—Rb4^i^	55 (5)	Rb4—Rb5—S2	77 (8)
Rb5^xii^—Rb4—Rb4^i^	57 (3)	Rb5^xii^—Rb5—S2	87 (3)
Rb5^xiii^—Rb4—Rb4^i^	123 (3)	Rb5^xiii^—Rb5—S2	128 (3)
Rb5—Rb4—Rb4^v^	90.000 (19)	Rb4^xii^—Rb5—S2	92.3 (10)
Rb4^x^—Rb4—Rb4^v^	55 (5)	Rb4^xiii^—Rb5—S2	123 (3)
Rb4^xi^—Rb4—Rb4^v^	125 (5)	Rb5^i^—Rb5—S2	62.8 (7)
Rb5^xii^—Rb4—Rb4^v^	123 (3)	Rb5^v^—Rb5—S2	117.2 (7)
Rb5^xiii^—Rb4—Rb4^v^	57 (3)	Rb4^v^—Rb5—S2	112 (3)
Rb4^i^—Rb4—Rb4^v^	180.00 (10)	Rb4^i^—Rb5—S2	59.7 (19)
Rb5—Rb4—Rb5^i^	73.1 (16)	S3^vi^—Rb5—S2	55.4 (8)
Rb4^x^—Rb4—Rb5^i^	140 (7)	S2^v^—Rb5—S2	54.4 (14)
Rb4^xi^—Rb4—Rb5^i^	71 (3)		

Symmetry codes: (i) *x*, *y*+1, *z*; (ii) −*x*, −*y*+1, −*z*+2; (iii) −*x*+1, −*y*+1, −*z*+2; (iv) −*x*, −*y*+2, −*z*+2; (v) *x*, *y*−1, *z*; (vi) −*x*+1, −*y*, −*z*+2; (vii) −*x*, −*y*, −*z*+2; (viii) *x*, *y*+1, *z*+1; (ix) *x*, *y*, *z*+1; (x) −*x*, −*y*−1, −*z*+1; (xi) −*x*, −*y*, −*z*+1; (xii) −*x*+1, −*y*, −*z*+1; (xiii) −*x*+1, −*y*−1, −*z*+1.
